# Two new species for *Gochnatia* Kunth (Asteraceae, Gochnatieae) and an extension of the tribal range into Ecuador

**DOI:** 10.3897/phytokeys.139.38354

**Published:** 2020-01-27

**Authors:** Harold Robinson, Vicki A. Funk

**Affiliations:** 1 US National Herbarium, Department of Botany, NMNH, Smithsonian Institution, Washington, D.C. USA Smithsonian Institution Washington United States of America

**Keywords:** Andes, *Moquiniastrum*, Compositae, South America, trichomes

## Abstract

Two new species are added to the narrowly delimited genus *Gochnatia*. Of these, *G.lojaensis***sp. nov.** represents a northern extension of the genus and tribe into Ecuador and *G.recticulifolia***sp. nov.** occurs in northern Peru. In addition to descriptions for the two new species, a key is provided for all known species in the genus *Gochnatia* and a pubescence character is noted that clearly separates *Gochnatia* from *Moquiniastrum*.

## Introduction

In the process of working on the treatment of the tribe Vernonieae (Asteraceae) for the Flora of Ecuador (Robinson and Funk 2018), an unidentified specimen that came in on loan from AAU was determined not to be Vernonieae. Although it seemed to belong to the tribe Gochnatieae J.Panero & V.A.Funk, that tribe had not been reported from Ecuador (Funk et al. 2014). In trying to identify the specimen, other Gochnatieae from US were examined and two more specimens were found that were questionable as to species. The first of these three specimens remained in a folder of “work to be done” for a number of years. As the loans were being annotated in preparation for their return, the folder reappeared. One of the three specimens was determined to be a slight variant of *Gochnatiaarequipensis* Sandwith. The remaining two specimens were more difficult to assign to a known species and eventually we decided they were new to science. The two species can now be described as true members of narrowly defined *Gochnatia* Kunth, a genus that is primarily of Central Andean distribution.

The genus *Gochnatia* was revised by Cabrera (1971) and subsequently it became the basis for a tribe (Panero and Funk 2002, 2007; Sancho and Freire 2009). The recent history of the basal tribes was summarized by Ortiz et al. (2009). Recent papers on the immediate group of genera include Katinas et al. (2008) and Funk et al. (2009, 2014). Generic level papers include those on *Richterago* Kuntze (Roque and Pirani 2001), *Anastraphia* D.Don (Ventosa-Rodríguez and Herrera-Oliver 2011), *Moquiniastrum* (Cabrera) G.Sancho (Sancho et al. 2013), and *Nahuatlea* V.A.Funk (Funk et al. 2017). These efforts have further refined our concepts. Some species that Cabrera included in the genus *Gochnatia* (s.l.) such as the Asian taxa are reinstated in their own genera (*Leucomeris* D.Don and *Nouelia* Franch.) and have been placed in a different tribe (Hyalideae) and are therefore, not part of the Gochnatieae. Others, like *Cnicothamnus* Griseb. and *Cyclolepis* Gillies ex D.Don had not been placed in *Gochnatia* s.l. by Cabrera (1971) and are now associated with the tribe. There are now 7 genera and 57 species in the tribe and *Gochnatia* s.s. now contains 10 species. In contrast, the tribe Hyalideae Panero, typified by the genus *Hyalis* D.Don ex Hook. & Arn., was placed in subfamily Wunderlichioideae and contained four genera: *Ianthopappus* Roque & D.J.N.Hind, *Hyalis* D.Don ex Hook & Arn., *Leucomeris*, and *Nouelia*, and a total of six species. Recent results from next generation sequencing (Mandel et al. 2019) demonstrated that the Hyalideae are now better placed in the Stifftioideae.

## Methods

Collections were studied from the following herbaria: AAU and US. Morphological characters were assessed and measured from herbarium material. Florets and fruit were rehydrated in water prior to dissection and measurement. Other characters were measured directly from the herbarium specimens. Some parts such as trichomes were mounted on slides in Hoyer’s solution (Anderson 1954). Corolla color, habit and habitat information were taken from the labels of the holotypes. Both species are only known from the type collection.

## Results

Two new species are described below: *Gochnatialojaensis* sp. nov. from the mid-elevations of southern Ecuador and *Gochnatiarecticulifolia* sp. nov. from northern Peru.

### Gochnatia lojaensis

Taxon classificationPlantaeAsteralesAsteraceae

H.Rob. & V.A.Funk sp. nov.

E4470E65-3794-59E4-9769-CFA5501DA010

urn:lsid:ipni.org:names:77204856-1

**Figure 1.**
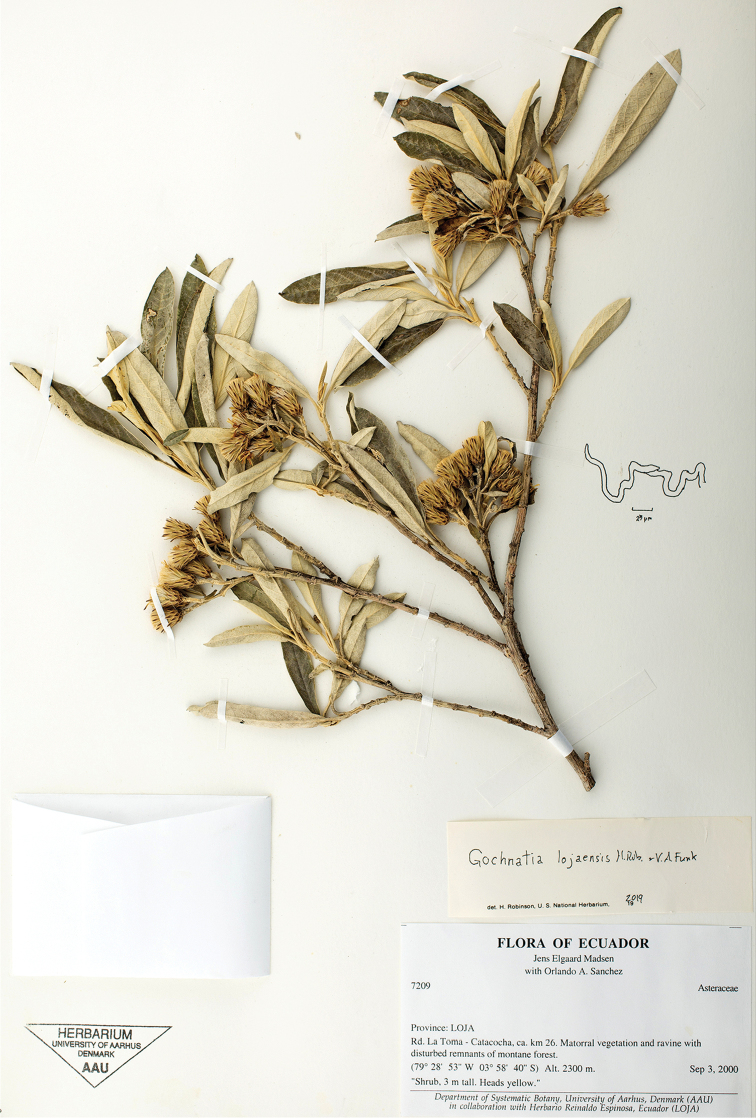
Holotype specimen (Madsen and Sanchez 7209, AAU) of *Gochnatialojaensis* H.Rob. & V.A.Funk, including drawing of obliquely capped tip of a contorted trichome from abaxial surface of leaf.

**Figure 2.**
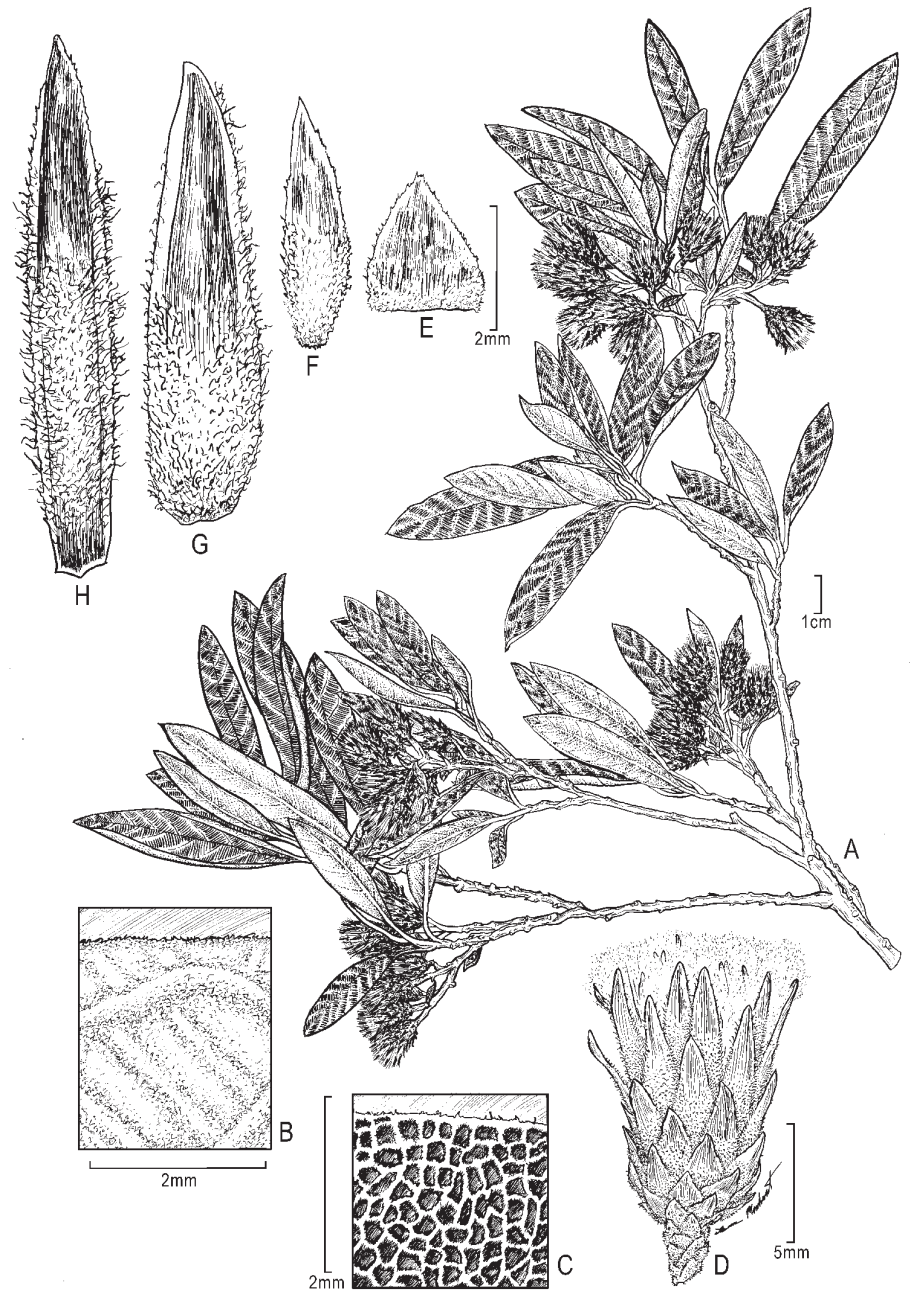
*Gochnatialojaensis***A** habit **B** abaxial surface of leaf showing density of pubescence **C** adaxial surface showing venation **D** capitulum **E–H** involucral bracts, inner, median and outer. Drawing by Lauren Merchant (US).

**Figure 3.**
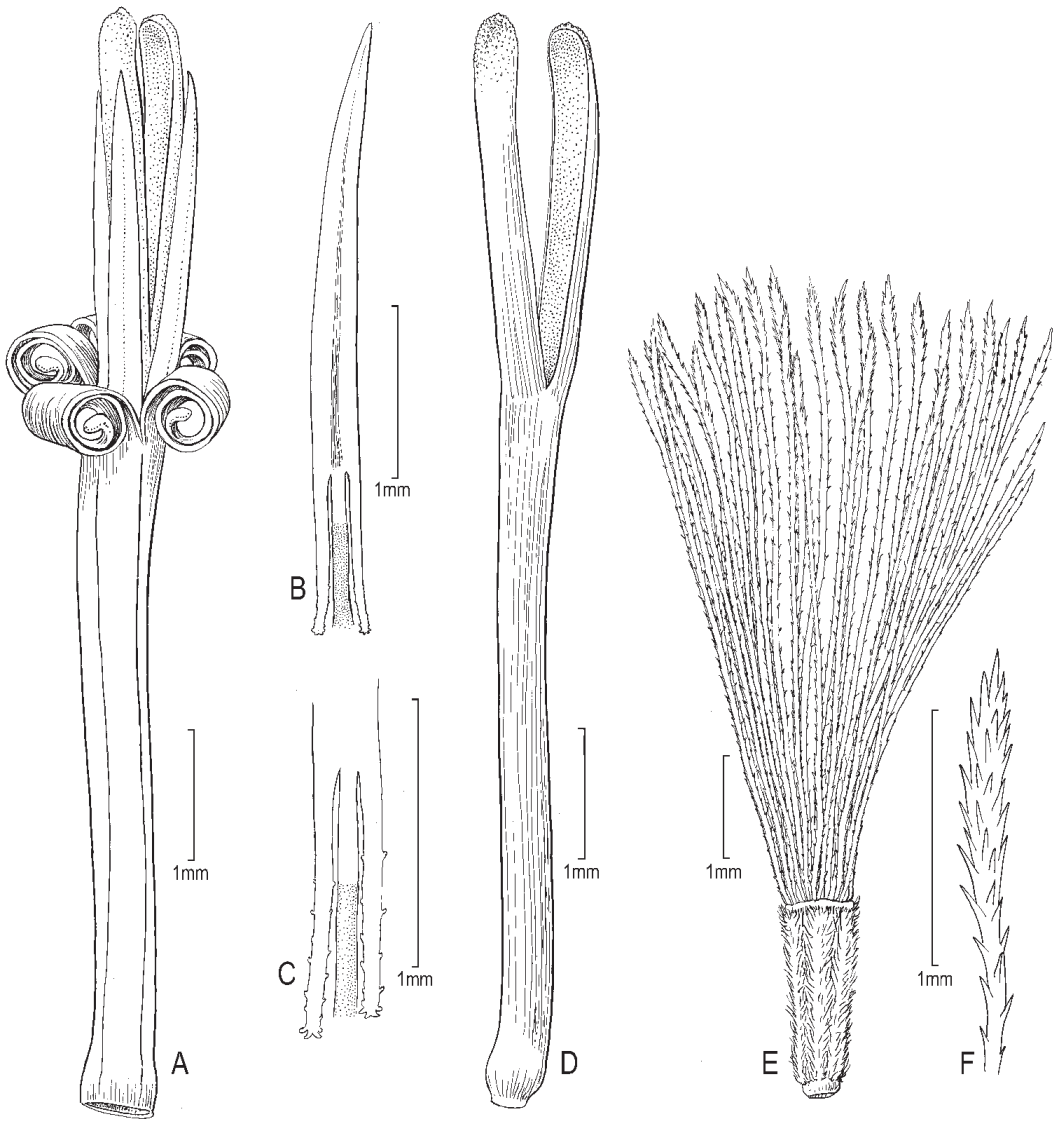
*Gochnatialojaensis*, floral details **A** corolla with backward rolled lobes and tips of anthers and style **B** anther with fringe of retrorse teeth near and on tips of basal spurs **C** enlargement of basal spur of anther **D** style **E** achene showing more densely scabrid tips of inner pappus bristles, sparse outer pappus not shown **F** enlargement of tip of inner pappus bristle.

Figs 1 , 2 , 3 , 5A , 5C

#### Type.

Ecuador. Prov. Loja: La Toma – Catacocha road ca. km 26, shrub 3 m tall, heads yellow, 79°28'53"W, 03°58'40"S, 2300 m alt., 3 Sep 2000, *Jens Elgaard Madsen with Orlandro A. Sanchez*, *7209* (holotype AAU!; isotypes, LOJA, US frag.!).

#### Description.

***Shrub*** to 3 m tall, with numerous branches. ***Stems*** grayish, wrinkled when dry; internodes 2–5 mm long, surface with yellowish-gray, evanescent, granular appearing pubescence, composed of tightly glomerulous contorted trichomes, pith solid, ca. 2 mm wide. ***Leaves*** spirally alternate, petioles 7–8 mm long; blades narrowly elliptical, 3–6.5 cm long, 0.9–1.4 cm wide, base obtuse, margins entire, plane, apex subacute, adaxial surface green with thin evanescent pubescence in young leaves, with minute reticulum of prominulous veinlets (veins slightly prominent), secondary veins spreading from midvein at ca. 45° angles; abaxial surface covered with dense yellowish tomentum of slender highly contorted trichomes, trichomes with few thin-walled cells at base, sometimes with slightly off-set apical cells separated by an oblique cross-wall, costa prominent to near leaf-tip. ***Inflorescence*** corymbiform, with clusters of 10–15 heads apical on leafy branches, cluster usually becoming over-topped by younger branches and longer leaves, with small bracteoles 1.0–1.5 cm. long among heads; peduncles 0.7–l.0 cm long, longer peduncles with minute scale-like bracteoles; involucres campanulate, 1–1.2 cm high, ca. 0.8–0.9 cm wide at anthesis, with ca. 50 subimbricate appressed bracts in ca. 6 series, bracts progressing from basal scales ca. 1.5 mm long and wide to many progressively longer lanceolate median bracts to few somewhat deciduous linear inner bracts ca. 9 mm long and 1 mm wide, outer surfaces of bracts glabrous on most exposed surfaces, castaneous, yellow along margins, bases of bracts with yellowish tomentum of slender contorted trichomes. Upper surface of receptacles glabrous, alveolate. ***Florets*** homogamous, ca. 15 per capitulum; corollas yellowish with darkened tips, glabrous outside, ca. 9 mm long, basal tube ca. 4 mm. long, throat 1 mm long, narrowly funnelform, lobes 3.5 mm long, linear, 0.4 mm wide, coiled backward at anthesis Fig. 3A), with protuberant slightly rugulose elongate cells inside; anthers ca. 2.5 mm long (Fig. 3B), basal tails lanceolate, ca. 1 mm long, with fringed with retrorse teeth near and at tips (Fig. 3C), apical appendage indurate, ca. 1 mm long, oblong-ovate with marked apical apiculus; pollen prolate, 30–35 µm diam. and 35–40 µm long; styles slightly broadened and blunt at tips. ***Achenes*** ca. 2.5 mm long, 5-costate (Fig. 3E), densely villosulous with ascending twin-hairs, hairs not cleft at tips usually with one cell longer than other; carpopodium annuliform; pappus of ca. 50 capillary bristles, whitish, mostly ca. 7 mm long, broader and more strongly barbellate near tips (Fig. 3F), some shorter outer bristles with slender tips.

#### Distribution and ecology.

Known only from the type collection which places it in “Matorral vegetation and ravine with disturbed remnants of montane forest.”

#### Conservation status.

DD (according IUCN 2019).

#### Etymology.

*Gochnatialojaensis* is named after the Ecuadorian province where it was collected.

#### Notes.

Distinguishing characteristics include the corymbiform clusters of numerous heads and the narrow castaneous involucral bracts with narrowly blunted tips.

The position of the new species was at first in doubt. It was near the geographic range of *Gochnatia* typified by *G.vernonioides* Kunth from Peru, but it had the more elongate leaves often associated with the presently recognized separate genus *Moquiniastrum*. A detailed study of the plant now confirms a position in *Gochnatia*: the pubescence is particularly indicative, being a thick tomentum and not the loose stalked T-shaped hairs common in *Moquiniastrum* (Fig. 5F). The hairs do show one interesting tendency toward the T-shaped form, with some hairs having an apical cell that is obliquely mounted on the longer contorted cell. Such an apical cell usually has the lower end slightly projecting, a sub-T-shaped specialization. This remains totally different from the well-developed T-shape form seen in *Moquiniastrum* (Fig. 5F).

### Gochnatia recticulifolia

Taxon classificationPlantaeAsteralesAsteraceae

H.Rob. & V.A.Funk sp. nov.

F23A126E-1B3B-5640-8E8E-09568B33B1FD

urn:lsid:ipni.org:names:77204857-1

Figs 4 , 5B , 5D

#### Type.

Peru. Department Ancash: Callejon de Huaylas, trail to cave across Río Santo [Río Santa] from Mancos, shrub to 3 m tall. Flowers yellow, phyllaries green. 9 April 1970, *C. Earle Smith Jr. & Jacinto Blas 4901* (holotype US!). Collected in co-operation with the Seasonal Transhumance and Preceramic Occupation of the Callejon de Huaylas Project – Thomas F. Lynch, Director and according to the label the Vernacular name is *Juancablanca*.

#### Description.

***Shrub*** to 3 m tall; stems gnarled, thickened and blackish near base, to ca. 8 mm wide, with scarcely noticeable narrow pith; younger stems as slender shoots, with internodes 3–10 mm long, covered with grayish tomentum. ***Leaves*** alternate, petioles ca. 4 mm long; laminae ovate oblong, mostly 1.6–2.5 cm long, 1.2–1.8 wide, flat or sometimes folded along midvein, base short-obtuse, apex usually rounded with slight mucro, margins flat, not recurved, entire, adaxial surface greenish with minute thin evanescent floccose puberulence, abaxial surface with grayish granular-looking pubescence consisting of slender highly contorted trichomes with few thin-walled cells at base, sometimes with slightly off-set apical cells separated by an oblique cross-wall, with a weakly prominulous midvein, 3 or 4 pairs of ascending, secondary veins, and a minute reticulum of veinlets evident on both surfaces that are not obscured by pubescence. ***Inflorescence*** of a solitary capitulum or 2–3 grouped together at tips of leafy stems; involucres broadly campanulate at anthesis, ca. 9 mm high and 11 mm diam., bracts ca. 40 in ca. 6 gradate series, basal bracts broadly ovate, 1.5–2.5 mm long, to 2.5 mm wide, rounded to obtuse at tips, inner bracts lanceolate, to ca. 7 mm long, acute, outer surface thinly pilosulous with weak indumentum near bases; receptacle slightly crested between areoles. ***Florets*** homogamous 35–40 per capitulum, corollas yellow, ca. 8–9 mm long, without long hairs outside, basal tube ca. 3.5 mm long, throat ca. 1.5 mm long, narrowly funnelform, lobes linear, ca. 3.5 mm long, ca. 0.2 mm wide; anther thecae ca. 1.8 mm long, tails ca. l.2 mm long, with a dense fringe of narrow hairs, apical appendage ca. 0.9 mm long, narrowly ovate with acuminate tip; pollen 30–40 µm in diam. and 50–55 µm long; styles slightly broadened and blunt at tips. ***Achene*** cylindrical, ca. 4 mm long, sericeous with slender setulae; pappus pale yellow with ca. 35 inner capillary bristles, up to 7 mm long, most with distinctly broader and more densely scabrous tips, and with numerous outer shorter weakly barbellate bristles of various lengths with slender tips.

**Figure 4.**
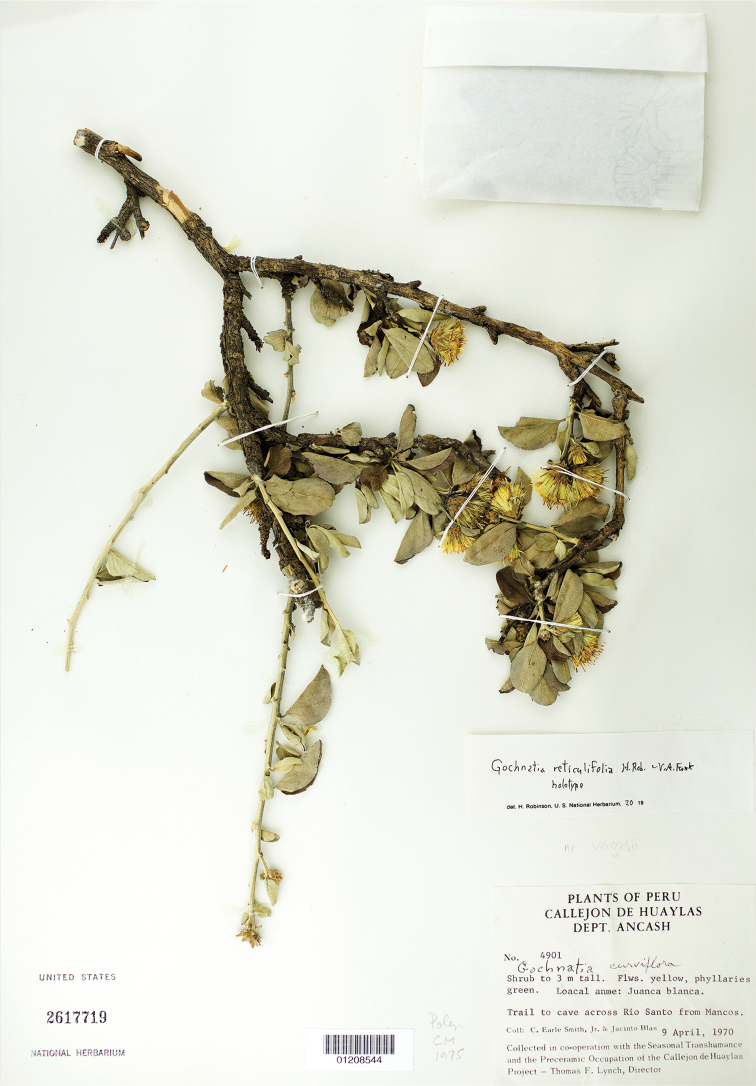
Holotype specimen (Smith and Blas 4901, US) of *Gochnatiarecticulifolia* H.Rob & V.A.Funk. http://n2t.net/ark:/65665/3285d4b9d-0bd0-421f-bc05-f0e82f454950

#### Distribution.

Known only from the type collection which places it in northern Peru.

#### Conservation status.

DD (according IUCN 2019).

#### Etymology.

The epithet for *Gochnatiarecticulifolia* is based on the minute reticulum of veinlets that is evident on both surfaces of the leaf.

#### Notes.

The type specimen was original identified in the herbarium as *G.curvifolia* S.F.Blake, a potentially related species of mostly Bolivian distribution. However, *G.curvifolia* has pointed leaf tips and a minute reticulum of the leaf veins mostly obscured by pubescence. The corollas of *G.recticulifolia* have no hint of the pilosity seen in many but not all specimens of *G.curvifolia* and the closely related *G.boliviana* S.F. Blake. *G.recticulifolia* may actually be closer to the unseen *G.vargasii* Cabrera of the Department of Apurimac in Peru, but the latter is distinct in having more acute leaves and serrate leaf margins.

**Figure 5.**
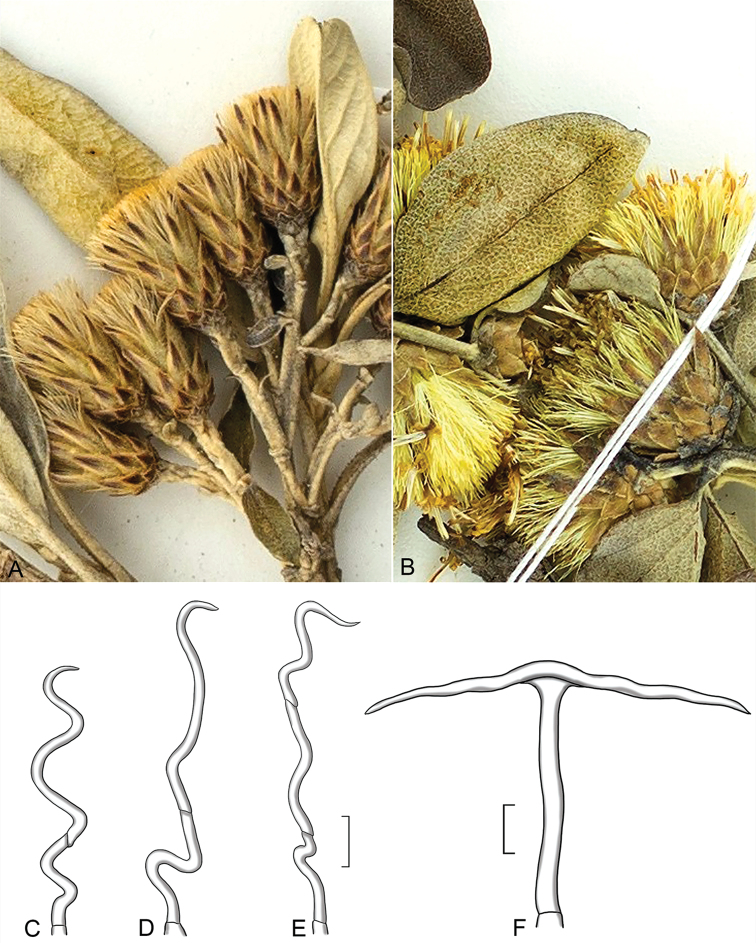
Enlargements of capitula, leaves and trichomes. Capitula and leaves **A***Gochnatialojaensis***B***G.recticulifolia***C–F** trichomes from abaxial surfaces of leaves of Gochnatieae: *Gochnatialojaensis* sp. nov. (**C**), *Gochnatiarecticulifolia* sp. nov. (**D**), *Gochnatiavernonioides* Kunth (from Ferreyra 7097, US) (**E**), http://n2t.net/ark:/65665/3ec557cf1-0267-44ee-8649-ee3170d4c7f2 and *Moquiniastrumpolymorphum* (Less.) (**F**) G. Sancho (from Ganev 1201-HUEFS 12265, US). http://n2t.net/ark:/65665/3d878282f-4ead-4312-9189-60a09f4a7f43; Scale bars: 30 µm.

As a result of our investigations we emphasize the trichome character that provides an additional distinction between *Gochnatia* s.s. and *Moquiniastrum*: the trichomes on the abaxial surface of the leaf. In *Gochnatia* they are slender, highly contorted trichomes with a few thin-walled cells at base, sometimes with slightly off-set apical cells separated by an oblique cross-wall (Fig. 5C–E). In *Moquiniastrum* the trichomes are T-shaped, often long-stalked with an elongate cap-cell attached at the mid-point (Fig. 5F). We examined the generitypes of both genera as well as our two new species. *Gochnatiavernonioides* (Fig. 5E), the generitype of *Gochnatia* has some trichomes with two such oblique cross-walls and off-set cells. Certainly, the distribution of this character should be examined for all 10 species in an upcoming monograph of *Gochnatia* s.s. (Sancho et al., in prep.).

The 10 species now recognized in *Gochnatia* can be distinguished by the following key. Many details are from Cabrera (1971), especially regarding *G.vargasii* of which proper material has not been seen.

##### Key to *Gochnatia* species

**Table:** 

1	Involucre greatly attenuated at base, with ca. 10 series of bracts of increasing size	***G.palosanto* Cabrera (Bolivia to Argentina)**
–	Involucre abruptly rounded at base, with only 5–7 tiers of bracts	**2**
2	Capitula narrow, with 7–12 florets; stems with small oblong to elliptical leaves often in axillary fascicles, mostly 5–20 mm long; petioles less than 2 mm long	**3**
–	Capitula campanulate, with more than 12 florets; leaves ovate, over 20 mm long, petioles 2–6 mm long	**4**
3	Involucre 8–9 mm high; capitula with 7–8 florets; corollas 7–9 mm long	***G.cardenasii* S.F. Blake (Bolivia)**
–	Involucre 10–15 mm high; capitula with 9–12 florets; corollas to 15 mm long	***G.arequipensis* Sandwith (Central Peru)**
4	Leaves with toothed margins	***G.vargasii* Cabrera (Apurimac, Peru)**
–	Leaves with entire margins	**5**
5	Lower bracts of involucre broadly ovate	**6**
–	Lower bracts of involucre lanceolate; corollas not sericeous outside on throat	**8**
6	Leaves with blunt or rounded tips, surfaces with thin granular appearing pubescence that does not obscure minute reticulum of veinlets; corolla with no evident pilosity on outer surface	***G.recticulifolia* (N Peru)**
–	Leaves with dense tomentum on at least abaxial surface, reticulum of veinlets obscured; corollas often with pilosity on outer surface of throat	**7**
7	Capitula with 12–20 florets; involucres 12–15 mm high, higher than wide at anthesis	***G.curviflora* (Griseb.) Hoffm. (S Bolivia to N Argentina)**
–	Capitula with at least 40 florets; involucres 12–15 mm high, almost as wide as high at anthesis	***G.boliviana* S.F.Blake (Central Bolivia)**
8	Capitula in corymbiform clusters; involucral bracts blunt at narrow tips; leaves lanceolate to narrowly oblong; spurs of anthers with only short teeth	***G.lojaensis* (S Ecuador)**
–	Capitula few at tips of leafy branches; involucral bracts often mucronate to acuminate at tips; leaves ovate; spurs of anthers with dense fringe of hairs	**9**
9	Involucres nearly as wide as high at anthesis, 10–11 mm high; involucral bracts shortly mucronate at tips; leaves to 80 mm long, 35 mm wide	***G.vernonioides* Kunth (N Peru)**
–	Involucres higher than wide, to 14–17 mm high; involucral bracts mostly mucronate to aristate at tips; leaves 10–23 mm long, 9–18 mm wide	***G.patazina* Cabrera (Central Peru)**

Excluded is *Gochnatialanceolata* Beltram & Ferreyra, with glabrous leaves. The species has been transferred to a new genus *Paquirea* (Panero and Freire 2013).

The gnarled appearance of the stems was found in the newly described *G.recticulifolia* and in one specimen of *G.arequipensis* Sandwith. The lack of this character in all other collections of *Gochnatia* may be an artifact of collecting and it may occur in other species but was omitted during the pressing of the plants.

## Supplementary Material

XML Treatment for Gochnatia lojaensis

XML Treatment for Gochnatia recticulifolia

## References

[B1] Anderson LE (1954) Hoyer’s solution as a rapid mounting medium for Bryophytes. The Bryologist 57(3): 242–244. 10.2307/3240091

[B2] Cabrera AL (1971) Revision del genero *Gochnatia*. Revista del Museo de La Plata, Nueva Serie. Sección Botánica 12(66): 1–160.

[B3] Funk VA Susanna A Stuessy T Bayer B (2009) Systematics, Evolution, and Biogeography of the Compositae. International Association for Plant Taxonomy (IAPT), Vienna.

[B4] Funk VA Sancho G Roque N Kelloff CL Ventosa-Rodríguez I Diazgranado M Bonifacino JM Chan R (2014) A phylogeny of the Gochnatieae: Understanding a critically placed tribe in the Compositae. Taxon 63(4): 859–882. 10.12705/634.27

[B5] Funk VA Sancho G Roque N (2017) *Nahuatlea*: A new genus of Compositae (Gochnatieae) from North America. PhytoKeys 91: 105–124. 10.3897/phytokeys.91.21340 PMC5769713 29362548

[B6] IUCN (2019) The IUCN Red List of Threatened Species. Version 2019-2. http://www.iucnredlist.org [Downloaded on 18.07.2019]

[B7] Katinas L Pruski JF Sancho G Tellería MC (2008) The subfamily Mutisioideae (Asteraceae). Botanical Review 74(4): 469–716. 10.1007/s12229-008-9016-6

[B8] Mandel JR Dikow RB Siniscalchi CM Thape R Watson LE Funk VA (2019) A fully resolved Backbone Phylogeny reveals numerous dispersals and explosive diversifications throughout the history of Asteraceae. Proceedings of the National Academy of Sciences of the United States of America 116(28): 14083–14088. 10.1073/pnas.1903871116 31209018 PMC6628808

[B9] Ortiz S Bonifacino JM Crisci JV Funk VA Hansen HV Hind DJN Katinas L Roque N Sancho G Susanna A Tellería MC (2009) The basal grade of Compositae: Mutisieae (sensu Cabrera) and Carduoideae. In: Funk VA Susanna A Stuessy T Bayer R (Eds) Systematics, Evolution, and Biogeography of Compositae.International Association for Plant Taxonomy (IAPT), Vienna, 193–213.

[B10] Panero JL Freire SE (2013) *Paquirea*, a new Andean genus for *Chucoalanceolata* (Asteraceae, Mutisioidea, Onoserideae). Phytoneuron 2013: 1–11.

[B11] Panero JL Funk VA (2002) Toward a phylogenetic subfamilial classification for the Compositae (Asteraceae). Proceedings of the Biological Society of Washington 115(4): 909–922.

[B12] Panero JL Funk VA (2007) New infrafamilial taxa in Asteraceae. Phytologia 89(3): 356–360.

[B13] Robinson H Funk VA (2018) Compositae-Vernonieae, Part 190 (1). In: Persson C, Eriksson R, Romoleroux K, Ståhl B (Eds) Flora of Ecuador (Vol. 94). University of Gothenburg, Gothenburg.

[B14] Roque N Pirani JR (2001) Reinstatement of the name *Richterago* Kuntze and recircumscription of the genus to include species formerly treated as *Actinoseris* (Endl.) Cabrera (Compositae, Mutisieae). Taxon 50(4): 1155–1160. 10.2307/1224734

[B15] Sancho G Freire SE (2009) Gochnatieae (Gochnatioideae) and Hyalideae (Wunderlichioideae p.p.). In: Funk VA Susanna A Stuessy T Bayer R (Eds) Systematics, Evolution, and Biogeography of Compositae.International Association for Plant Taxonomy (IAPT), Vienna, 249–260.

[B16] Sancho G Funk VA Roque N (2013) *Moquiniastrum* (Gochnatieae, Asteraceae): Disentangling the paraphyletic *Gochnatia*. Phytotaxa 147(1): 26–34. 10.11646/phytotaxa.147.1.3

[B17] Ventosa-Rodríguez I Herrera-Oliver PP (2011) Restoration of the name *Anastraphia* to define the species in the section Anastraphioides of *Gochnatia* (Gochnatioideae, Asteraceae). Compositae Newsletter 49: 23–37.

